# From Cultivation to Commerce: A Comprehensive Review of the Ecology, Chemistry, Production, Sustainability, and Market Prospects of an Ethiopian Herb Korarima (*Aframomum corrorima*)

**DOI:** 10.1155/sci5/9532686

**Published:** 2026-06-29

**Authors:** Netsanet Tena, Bizuayehu Desta

**Affiliations:** ^1^ Department of Plant Science, College of Agriculture and Natural Resource Sciences, Debre Berhan University, P.O. Box 445, Debre Berhan, Ethiopia, dbu.edu.et; ^2^ Department of Horticulture, College of Agriculture and Natural Resource Sciences, Debre Berhan University, P.O. Box 445, Debre Berhan, Ethiopia, dbu.edu.et

**Keywords:** agronomic practices, Ethiopian spice, genetic resource decline, korarima (*Aframomum corrorima*), medicinal and culinary uses, sustainable production

## Abstract

Korarima *(Aframomum corrorima)* is a perennial aromatic herb native to Ethiopia, widely valued for its culinary, medicinal, and cultural importance. It naturally grows under shaded forest ecosystems; however, deforestation and habitat degradation have led to a significant decline in its wild populations. Despite its economic importance, consolidated information on its production systems, ecology, chemical properties, and market potential remains limited. This review systematically synthesizes existing knowledge on korarima by focusing on four key areas: its ecological requirements and distribution, agronomic and production practices, chemical composition and quality attributes, and its economic importance, including household income contribution, trade potential, and value chain characteristics. In addition, the review highlights major production and utilization constraints such as genetic erosion, limited agronomic technologies, and weak market development. The paper also outlines future prospects for sustainable production, conservation, and improved utilization of korarima as a high‐value Ethiopian spice, emphasizing its role in biodiversity conservation and rural livelihood enhancement.

## 1. Introduction

Ethiopia is endowed with remarkable agroecological diversity that supports the production of a wide range of spice crops with substantial economic, cultural, and culinary importance [1, 2]. The country is recognized as one of the leading spice‐producing nations in East Africa, with approximately 222,700 ha of land currently under spice cultivation and an annual production exceeding 240,000 tons [3]. In addition, more than 50 spice species are cultivated across diverse agroecological zones, and there is considerable potential to expand spice production into an additional 200,000 ha, particularly in lowland areas. Among these species, korarima (*Aframomum corrorima*), commonly known as Ethiopian cardamom, is one of the most valuable indigenous spices due to its unique aroma, multipurpose uses, and high market demand [4].

Korarima is a perennial aromatic herb belonging to the family Zingiberaceae and is closely related to true cardamom (*Elettaria cardamomum*) [5]. It occurs naturally in the moist evergreen forests of southern and western Ethiopia and is also cultivated in several highland areas, including regions around Lake Tana and Gelemso [6–8]. The dried seeds of korarima are widely used as a spice in traditional Ethiopian cuisine, particularly in flavoring dishes and spice mixtures such as *berbere* and *mitmita*, as well as in beverages, including coffee [9]. Beyond its culinary value, korarima has long been recognized in traditional Ethiopian medicine for treating various human and livestock ailments, reflecting the country’s extensive reliance on herbal remedies. It is widely reported that a large proportion of the Ethiopian population (around 80%) uses traditional medicinal plants as part of primary healthcare, particularly in rural areas where access to modern medical services is limited [10–12].

In addition to its socioeconomic importance, korarima plays a significant ecological role. When cultivated under shaded agroforestry systems, it contributes to soil conservation, reduces erosion, and supports forest biodiversity, thereby linking livelihood improvement with environmental sustainability [13, 14]. Economically, Ethiopia exports dried korarima pods to several regional and international markets, including Sudan, Egypt, the Arabian Peninsula, Iran, India, and parts of Europe, highlighting its growing global relevance [6, 15].

Despite its importance, korarima production remains constrained by several challenges, including habitat degradation, deforestation, lack of improved varieties, and poor agronomic management practices. These constraints have resulted in declining natural populations, unstable yields, and genetic erosion of the species [7, 16, 17]. Furthermore, existing research on production systems, value chain development, and sustainable management of korarima remains fragmented, limiting the development of evidence‐based interventions.

Therefore, this review aims to synthesize existing knowledge on korarima production, utilization, and constraints, with particular emphasis on identifying key production bottlenecks and research gaps. It also highlights critical areas for improvement in agronomic practices, genetic resource conservation, and value chain development. By doing so, the review provides a comprehensive reference for researchers, extension workers, policymakers, and stakeholders engaged in spice development and agroforestry systems in Ethiopia and beyond.

## 2. Ecological Requirements of Korarima

Ethiopia provides a wide range of agroecological zones highly favorable for the production of spice crops. Korarima, a spice species indigenous to the country, occurs naturally in forest ecosystems and is also maintained as a valuable component of household gardens, particularly in southern Ethiopia [18, 19]. This perennial species thrives at elevations of approximately 1350–2000 m above sea level [18, 19], typically in moderately shaded to semiopen environments within montane rainforest systems.

Optimal growth is achieved under mean annual temperatures of around 20°C and total annual rainfall ranging from 1300 to 2000 mm, with a relatively uniform distribution and no pronounced dry season [20]. During the main rainy period (June–August), korarima benefits from receiving about 50%–60% of its annual precipitation, which supports vegetative growth and reproductive development. The species grows across a range of soil textures, including clay, clay loam, loamy sand, sandy clay loam, and sand; however, it performs best in well‐drained, slightly acidic soils (pH 5.5–6.5). Deep to moderately deep soils (50–150 cm) rich in organic matter and essential nutrients are particularly suitable. Continuous soil moisture is critical for optimal growth, although prolonged waterlogging negatively affects root development and plant vigor [20].

In terms of nutrient requirements, korarima responds positively to balanced macronutrient availability, particularly nitrogen (N), phosphorus (P), and potassium (K), which support vegetative growth, root development, and capsule formation, respectively. Although the crop is often grown under low‐input conditions, the addition of organic amendments such as compost or farmyard manure can significantly enhance soil fertility and productivity. Micronutrients such as manganese (Mn) and zinc (Zn) also contribute to overall plant health, particularly in acidic forest soils where nutrient imbalances may occur [21].

Light availability plays a crucial role in korarima growth and yield. The crop is shade‐tolerant and performs best under partial shade (approximately 40%–60%), typically provided by forest canopies or agroforestry systems. Excessive sunlight exposure can lead to moisture stress and reduced productivity, while dense shade may limit flowering and capsule development. Similarly, korarima is sensitive to strong wind exposure, which can damage leaves, disrupt flowering, and reduce pollinator activity; thus, sheltered environments or forest‐based systems are most suitable. Pollination is another important ecological factor influencing yield. Korarima flowers are primarily pollinated by insects, particularly bees, and successful pollination is essential for capsule formation. Therefore, maintaining biodiversity and minimizing disturbances to pollinator populations are critical for sustained production [22, 23].

Weed competition can significantly affect early plant establishment and nutrient availability. Although mature plants can suppress some weeds due to canopy cover, regular weeding during the initial growth stages is necessary to reduce competition for light, nutrients, and moisture. Mulching with organic materials is often practiced to conserve soil moisture and suppress weed growth. Korarima is generally sensitive to salinity and does not perform well in saline or highly alkaline soils. Elevated salt levels can impair nutrient uptake, reduce growth, and ultimately lower yield. Consequently, the crop is best suited to humid, nonsaline environments with stable soil conditions. Overall, recent observations indicate particularly strong growth and productivity in the southwestern highlands of Ethiopia, where these ecological requirements—adequate rainfall, moderate temperature, fertile soils, and shaded environments—are naturally fulfilled [5].

## 3. Chemical Composition of Korarima Essential Oils

Korarima seeds have a mild, sweet flavor and are less peppery or pungent than the seeds of *Aframomum melegueta* K.Schum. (grain of paradise). The seeds contain essential oil, which has a typical odor and is sometimes called “nutmeg‐cardamom.” After distillation of dried comminuted fruits, 3%–3.5% of a pale yellow volatile oil with a flat cineolic odor can be obtained, and the following compounds have been found (all monoterpenes with the approximate amounts of the major ones): 1,8‐cineol 32%–35%, limonene 7%–14%, β‐pinene 4%–7%, sabinene 7%–9%, terpinen‐4‐ol 3%–5%, geraniol 5%, P‐cymene 4%, α‐pinene, α‐terpineol, and γ‐terpinene 3% each. Sesquiterpenes were identified in another analysis; the total composition was dominated by about 75% monoterpenes, including 1,8‐cineol (38%) and terpinyl acetate (11%), and 17% sesquiterpenes, including nerolidol (11%–14%), β‐caryophyllene (2%), and caryophyllene oxide (1%) [24].

The bioactive potential of korarima is broad. Several studies have demonstrated its antioxidant properties, largely attributed to its high monoterpene and sesquiterpene content, which can scavenge free radicals. The plant exhibits antibacterial activity against common Gram‐positive and Gram‐negative bacteria, and preliminary in vitro studies suggest antiproliferative effects on certain cancer cell lines. These pharmacological properties support its traditional use in herbal medicine for treating digestive disorders, infections, and inflammation [25, 26].

To characterize korarima’s chemical composition, various extraction and analytical techniques are employed. Essential oils are typically obtained through hydrodistillation or steam distillation, while solvent extraction (using methanol, ethanol, or hexane) is used for broader phytochemical profiling, including flavonoids, phenolic acids, and other secondary metabolites. The qualitative and quantitative composition of oils and extracts is commonly determined using gas chromatography–mass spectrometry (GC–MS) for volatile compounds and high‐performance liquid chromatography (HPLC) or ultra‐performance liquid chromatography (UPLC) for nonvolatile bioactive constituents. These techniques enable detailed profiling of both flavor‐related metabolites and bioactive compounds, providing a foundation for both culinary and medicinal applications of the spice [25, 26].

## 4. Importance of Korarima

### 4.1. Economic Importance

Korarima, often referred to as Ethiopian cardamom, represents a significant native cash crop that supports both domestic trade and export markets [27]. The dried fruit capsules are valued not only for local culinary applications but also as a high‐value export commodity. Historically, Ethiopia gained recognition as a significant supplier of korarima to global markets, where it was commonly utilized as a substitute for Indian cardamom (*Elettaria cardamomum*) [13, 14]. The economic importance of korarima as a cash crop has been increasingly recognized in specific production regions of Ethiopia, particularly in the southern and southwestern areas (e.g., the South Omo and South Ari districts). Empirical studies and value chain analyses indicate that korarima contributes to household income through the sale of dried capsules in local and regional markets, thereby supporting livelihood diversification among smallholder farmers [28].

Today, Ethiopia continues to ship korarima capsules to regional and overseas destinations such as Sudan, Egypt, Saudi Arabia, Iran, India, and parts of Scandinavia, where it remains a favored alternative to true cardamom. Despite this demand, export volumes have fluctuated considerably over recent decades due to inconsistent production and market conditions [24]. Studies in southern Ethiopia suggest that korarima can generate relatively high financial returns per hectare compared to several staple cereal crops, particularly under smallholder low‐input production systems. This is largely attributed to its high market value, suitability for low‐input agroforestry production systems, minimal dependence on external agricultural inputs, and strong demand in local and regional spice markets.

However, these findings are context‐specific and are based on economic analyses conducted under particular conditions, including forest‐shade or semishade production systems, local farm‐gate pricing, and limited use of purchased inputs such as fertilizer and improved planting materials [5]. Return estimates are typically derived using gross margin or return on investment (ROI) approaches that account for local production costs, labor inputs, and prevailing market prices, rather than standardized national‐level comparisons across cereal systems.

Recent economic analyses in southern Ethiopia indicate that profitability in korarima production is strongly influenced by labor availability, extension access, and market linkages, with notable variations across production areas [29]. Complementary studies on korarima production and value chains further demonstrate that labor availability, market access, and postharvest handling practices play a significant role in enhancing productivity and market participation [30]. Similarly, value chain analyses show that smallholders participate in multiple market outlets (collectors, retailers, and wholesalers), and their income gains depend strongly on processing, quality control, and access to price information [28]. In practice, farmers capture added value by engaging in postharvest activities such as proper drying, cleaning, and grading of capsules, which improve quality and price. However, constraints such as weak market organization, limited extension support, and poor access to infrastructure reduce their bargaining power and limit full value realization [31].

Korarima plays an important economic role in Ethiopia as both a domestic spice crop and an export commodity. The crop provides income for smallholder farmers, traders, processors, and local spice markets, particularly in southwestern Ethiopia. Studies have shown that korarima contributes significantly to rural livelihoods through cash income generation and employment opportunities along the value chain. Ethiopia has historically exported dried korarima capsules and seeds as a substitute for Indian cardamom due to their similar aromatic characteristics and lower production cost, and the crop can be cultivated under low‐input agroforestry systems, requires relatively lower external inputs, and is adaptable to shaded forest environments [17, 23]. These characteristics reduce production costs while supporting biodiversity conservation and sustainable land use. Moreover, its unique flavor profile and increasing recognition as “Ethiopian cardamom” create opportunities for niche premium markets [32].

The value chain analysis conducted in southern Ethiopia demonstrated that collectors, wholesalers, and retailers constitute the major market channels, accounting for 82.2%, 73.6%, and 35.5% of marketed korarima, respectively [28]. The crop is considered a high‐value shade‐grown spice with strong market demand in culinary, medicinal, and flavoring industries. In addition to local consumption, korarima possesses export potential because of increasing international interest in natural spices and Ethiopian traditional products. However, poor postharvest handling, lack of market standardization, weak branding, and limited export promotion continue to constrain its full economic potential.

Traditionally, the spice held cultural and economic value beyond its role in trade.

In Ethiopia and Eritrea, korarima fruits were once exchanged as a form of currency in barter transactions [33]. In some Arab countries, strings of dried fruits have occasionally been fashioned into ornamental necklaces or used as rosaries. Major importing nations also employ korarima as a flavoring agent for alcoholic beverages, confectionery, and cosmetic products, illustrating its versatile economic potential [27].

### 4.2. Ecological Importance

Korarima naturally thrives in forested ecosystems, where it is often harvested from semiwild stands. Cultivation of the spice in shaded, moist environments supports the conservation of endangered forest habitats in southern Ethiopia by encouraging farmers to maintain tree cover [34]. The plant’s dense growth also protects soil on sloping land, reducing erosion and promoting long‐term landscape stability [21]. As reported by Woldeyes [35], farmers managing korarima gardens are less inclined to remove the trees that shelter this valuable crop, thereby contributing to sustainable forest management. Beyond traditional garden settings, korarima also benefits wetlands; its ground cover limits evaporation and helps maintain streamflow, providing both ecological services and supplemental household income [18].

### 4.3. Culinary and Medicinal Importance

Korarima holds a central place in Ethiopian cuisine. Ground seeds are blended with other spices to flavor traditional sauces (wot), specialty breads, butter, and coffee, where they function as both a flavor enhancer and a natural preservative [13, 21, 36]. The fleshy aril surrounding the seeds is also consumed in some regions, adding to the plant’s culinary versatility. In southern Ethiopia, various plant parts, including seeds, pods, leaves, rhizomes, and flowers, are widely used in traditional medicine to treat a range of human and livestock ailments, particularly swellings of unknown origin [5]. These plant materials provide vitamins and minerals [5, 6, 25] and contain 1%–2% essential oils with a distinctive aroma. The oils are traditionally administered as tonics, laxatives, carminatives, and purgatives [24]. Laboratory studies have demonstrated strong antifungal activity in extracts from the pods, while leaves and rhizomes exhibit comparatively weaker inhibition and show no activity at low concentrations.

## 5. Cultivation Practices

### 5.1. Propagation Methods of Korarima

Korarima can be multiplied either from seed or by vegetative clump division. While seed propagation is possible, it is often constrained by strong seed dormancy, irregular germination, and slow seedling growth, making it less reliable for commercial cultivation. Vegetative propagation using rhizome segments or clumps is faster and ensures uniform, true‐to‐type plants. Seed propagation is often difficult because the species exhibits strong dormancy, whereas planting rhizome segments is faster and easier. However, heavy reliance on vegetative cuttings can damage productive stands and limit the supply of planting material for large‐scale expansion. To maintain mother plants and enable wider distribution, farmers frequently combine both methods. During peak harvest, fully ripened capsules are carefully selected, the seeds extracted, and then treated with ash to promote drying, inhibit fungal growth, and simplify handling during sowing [23]. Recent advances in micropropagation provide a complementary approach, ensuring an ample supply of disease‐free planting material for commercial production and supporting future improvement programs through tissue culture, genetic engineering, and germplasm conservation [37].

#### 5.1.1. Propagation by Rhizomes or Clumps

The traditional practice for multiplying korarima relies on splitting rhizomes and planting 1‐year‐old rhizome pieces or young suckers. This method accelerates early vegetative growth and guarantees offspring that remain true to type, but the availability of planting materials is limited, and productive mother plants may be sacrificed when large areas must be established [13, 14]. For effective rhizome propagation, farmers typically select healthy, mature rhizomes weighing 20–50 g, each with at least —two to three active buds. These rhizomes are planted at a depth of 5–10 cm in well‐drained, fertile soils rich in organic matter. Optimal spacing is generally 30–50 cm between plants within rows and 60–80 cm between rows, depending on terrain and management practices. Shaded or semishaded environments (approximately 40%–60% canopy cover) with moderate moisture and temperatures around 18°C–25°C provide favorable conditions for rhizome establishment and early growth. Soils should be slightly acidic to neutral (pH 5.5–6.5) to support root development, while prolonged waterlogging should be avoided to prevent rhizome rot.

Although vegetative propagation accelerates crop establishment, excessive removal of rhizomes from mother plants can reduce productivity and deplete planting stock, limiting large‐scale expansion. To mitigate this, farmers often combine seed and rhizome propagation, reserving part of the mother clumps for future planting while using seeds for wider distribution. During the harvest of mature capsules, fully ripened fruits are collected, seeds extracted, and treated with ash or sun‐drying to reduce fungal contamination and facilitate handling during sowing [23].

#### 5.1.2. Propagation by Seed

Seed propagation involves the germination of the embryo from within the hard seed coat. This process is hindered by both external and internal dormancy mechanisms.

External dormancy is largely mechanical, caused by the impermeable seed coat, whereas internal dormancy may be due to chemical inhibitors present in the endosperm [38–41]. Korarima seeds are capable of germination when sown as soon as possible after harvesting, but thereafter, due to the presence of dormancy, they lose their viability quickly [42]. Therefore, with prolonged storage time of more than 2 weeks, a decline in seed germination percentage and, consequently, variable and poor seedling development is a common phenomenon. Without seed treatment, korarima seeds need 40–45 days for germination [43]. However, seed treatment before sowing is believed to alleviate the problems associated with korarima seed propagation. Even after presowing seed treatment, for effective propagation of planting materials or production of seedlings in nurseries, attention should be given to nursery management practices, such as the crop’s water requirements at early stages of growth and development, the nature of germination and growth media in the seed bed, and the use of mulch to reduce water loss through evaporation, suppress weed growth, and maintain optimum soil temperature. The entire plantation, particularly the soil surface at the plant base, should be covered with mulch. To overcome seed dormancy problem, Eyob [13] reported that the most effective treatment for the highland cultivar “Mume” was a two‐step process: soaking seeds in 50% H_2_SO_4_ for 60 min followed by a 24‐h soak in 250 mg L^−1^ GA_3_, which significantly improved germination and seedling vigor.

### 5.2. Shade Management

Korarima is a shade‐loving perennial that requires careful management of canopy cover to maintain optimal growth, yield, and quality. Research at Jimma Agricultural Research Center (JARC) demonstrated that maintaining 55%–63% shade produces the highest yields by creating a microclimate that moderates soil temperature and conserves soil moisture [7]. Under these conditions, soil temperatures are typically maintained between 18°C and 22°C, which prevents stress to the shallow rhizome‐based root system, while soil moisture levels of approximately 60%–70% of field capacity support root development and vegetative growth without causing waterlogging.

Shade enhances korarima productivity by reducing evaporation, minimizing temperature fluctuations, and promoting organic matter decomposition, which improves soil fertility. The resulting microclimate also suppresses weed growth, reducing the need for labor‐intensive management practices such as frequent weeding and tillage [44]. In plantation systems, temporary shade species such as *Sesbania sesban* or *Grevillea robusta* are often used initially and then gradually replaced by permanent, broad‐leaved trees that retain foliage year‐round, providing consistent protection even during dry seasons.

Experimental studies have further highlighted the benefits of controlled shade. For instance, korarima plants grown under artificial blue plastic shade achieved the largest leaf area (4.22 m^2^), the greatest fresh weight (3766.4 g), and the highest dry weight (394.9 g), demonstrating the positive impact of moderated solar radiation on vegetative growth. These findings suggest that both natural and artificial shade can be effectively managed to optimize growth, provided that soil temperature and moisture remain within suitable ranges. Yield assessments under shaded conditions also show significant differences based on propagation method. Dry capsule yields of 21.8 q ha^−1^ from seed‐propagated plants and 15.2 q ha^−1^ from clump‐propagated plants indicate that propagation techniques interact with shade management to influence productivity [45].

Traditionally, korarima is cultivated in home gardens beneath native trees and often coexists with wild coffee (*Coffea arabica*), which provides natural canopy cover. However, deforestation, urbanization, and land‐use changes have reduced the availability of permanent shade trees, contributing to declining production and contraction of cultivation areas [7, 46]. These challenges underscore the importance of implementing managed shade systems, including both temporary and permanent species, to maintain the microclimatic conditions necessary for sustainable korarima production.

Yield variability in korarima is often attributed to “traditional farming practices”; however, this term encompasses several distinct management approaches with different implications for productivity and quality. In major producing areas of southern and southwestern Ethiopia, korarima is commonly cultivated under natural forest canopies or semimanaged shade systems, where excessive shading can reduce flowering intensity and capsule development [5, 47]. The crop is also frequently intercropped with perennial species such as coffee and enset, which may lead to competition for light, nutrients, and soil moisture, thereby affecting yield performance [48].

In addition, farmers typically use low planting densities and rely on minimal or no external inputs, particularly inorganic fertilizers, which limits soil nutrient availability and plant vigor under continuous production systems [5]. Traditional harvesting practices, including harvesting capsules at mixed maturity stages, and postharvest handling methods, such as sun‐drying on bare ground or simple mats, may further contribute to quality deterioration and postharvest losses [47]. While these practices are well adapted to local agroecological and low‐input farming systems, they collectively contribute to variability in both yield and quality. Therefore, distinguishing among these specific management components is essential for designing targeted interventions aimed at improving korarima productivity and postharvest quality.

In a study conducted by Eyob (52), Plants were grown under different colored plastic shade treatments (red, green, blue, and clear) and compared with an unshaded control under field conditions. Leaf area was measured at 300 days after planting (DAP), while fresh and dry weights were recorded at 360 DAP. Fresh weight represents total aboveground biomass immediately after harvest, and dry weight represents biomass after oven drying to constant weight. Values are means per plant. The least significant difference (LSD) at the 5% probability level is provided to compare treatment means within each column (Table 1).

**TABLE 1:** Effect of colored plastic shade treatments on leaf area, fresh weight, and dry weight per plant (source from: [49]).

Treatment	Leaf area (m^2^) 300 DAP	Fresh wt (g) 360 DAP	Dry wt (g)
Red plastic	3.31	2982.5	231.4
Green plastic	3.80	3075.1	262.0
Blue plastic	4.22	3766.4	394.9
Clear plastic	2.13	2029.2	193.8
Control	1.62	1633.9	112.7
LSD	0.23	351.1	51.2

### 5.3. Harvesting

Korarima, a spice native to Ethiopia, requires only minimal processing after harvest but careful attention to timing and handling to preserve quality. The crop exhibits an irregular flowering pattern similar to that of cardamom, with flowers and mature red fruits often appearing simultaneously on the same plant. Productive stands typically reach their first harvest about 3 years after planting and can remain commercially viable for approximately seven additional years [13, 17]. Fruit maturation occurs roughly 2–3 months after flowering, though exact harvesting periods vary among Ethiopian regions depending on rainfall patterns and temperature regimes. In most areas, flowering occurs from June to July, followed by harvesting between October and November.

At maturity, the fruit capsules change from green to bright red before turning dark brown after drying. For premium quality, capsules should be harvested when fully red and ripe.

Seeds inside mature capsules should appear dark brown and emit a characteristic pungent aroma when crushed. To maintain essential flavor compounds and prevent microbial contamination, harvesters should avoid damaging the capsules or creating openings during collection.

Postharvest processing involves sun‐drying the fruits on clean cemented platforms or elevated beds, with frequent turning to ensure uniform drying and to protect against mold or fungal infection [18].

### 5.4. Processing

Korarima capsules collected from natural forests in southern and southwestern Ethiopia are typically prepared for market using traditional drying techniques. A survey conducted near Bonga Jimma Research Center (JRC) documented several sequential steps employed by farmers to maintain product quality [50]. Immediately after harvest, capsules undergo a short predrying phase in which they are kept in warm, protected areas and covered with materials such as straw or enset leaves for approximately 10–15 days.

Two principal drying methods are then applied. Sun‐drying involves spreading the capsules on clean ground or specially prepared surfaces for 10–15 days, with the duration depending on the intensity and duration of daily sunlight. Smoke‐drying, in contrast, requires placing the capsules on wooden racks positioned above chimneys inside a roofed structure for 15–20 days, where gentle smoke and warmth facilitate moisture removal.

Korarima capsules were harvested at three maturity stages: mature green (MG), mature semired (MS), and mature deep red (MR). Seeds were extracted and dried using three drying structures (a cement floor, ground surface, and wire mesh) for 10, 15, and 20 days under ambient field conditions. Essential oil content (%) was determined on a dry weight basis after drying. Values represent the mean essential oil content. Means followed by different superscript letters within a column are significantly different at the 5% probability level based on the LSD test. Grand mean, LSD, and coefficient of variation (CV) are provided (Table 2).

**TABLE 2:** Interaction effects of harvesting stage, drying structure, and drying duration on essential oil content of dried korarima seeds (source from [51]).

Drying structures	Harvesting stages by drying durations								
	Mature green (MG)			Mature semired (MS)			Mature deep red (MR)		
	10 days	15 days	20 days	10 days	15 days	20 days	10 days	15 days	**20 days**
Cement	3.833^ijk^	3.133^l^	2.816^m^	4.050^fghi^	3.050^lm^	3.050^lm^	4.283^ef^	3.200^l^	3.200^l^
Ground	5.116^b^	4.533^d^	4.033^ghi^	4.866^c^	4.283^ef^	3.766^jk^	4.450^de^	4.200^fg^	3.683^k^
Wire mesh	5.533^a^	5.116^b^	4.533^d^	5.350^ab^	4.583^d^	4.116^fgh^	4.866^c^	4.283^ef^	3.950^hij^
	Grand Mean = 4.144			LSD (0.05) = 0.242			CV (%) = 3.567		

*Note:* Values denote mean essential oil percentage content (%) of dried korarima (*Aframomum corrorima*) seeds. Mean values that are not sharing common letters within rows and between rows are significantly different at 5% using Fisher’s LSD test. LSD (0.05) = least significant difference at 5% level of probability. The data have been subjected to an analysis of interaction among harvest stage, drying system, and drying time. Source: Adapted from [51].

Abbreviations: CV (%) = coefficient of variation, MG = mature green, MR = mature deep red, MS = mature semired.

Drying conditions significantly affect the chemical profile of the final product. For example, capsules harvested at the MG stage and dried for 10 days on wire mesh retained the highest essential oil content in their seeds, whereas those dried for 20 days on a cemented floor exhibited the lowest essential oil levels. These findings underscore the importance of selecting appropriate drying methods to preserve both aroma and market value [51].

## 6. Challenges and Future Prospects of Korarima Production in Ethiopia

The production of korarima in Ethiopia faces several agronomic and market‐related challenges that affect both productivity and profitability. One of the major constraints is limited access to soil fertility inputs, particularly inorganic fertilizers such as urea and blended NPS/NPK formulations, which are often unavailable or unaffordable for smallholder farmers. In many cases, farmers also apply insufficient amounts of organic amendments such as farmyard manure and compost due to labor limitations and competing household priorities. Studies from major producing areas indicate that inadequate fertilizer supply and soil fertility decline are among the key factors negatively affecting korarima productivity [5]. Similarly, the availability of quality planting materials is a critical bottleneck. Farmers predominantly use locally exchanged rhizomes or clump divisions, which are often not disease‐free and are not improved or certified materials. This contributes to poor field establishment and uneven productivity across farms [5, 52].

Market price stability for korarima is not defined by a fixed value but rather by the degree of seasonal fluctuation and predictability of farm‐gate prices. Empirical evidence from value chain studies in southern Ethiopia shows that prices vary significantly between harvest and off‐season periods due to supply concentration, weak market integration, and limited bargaining power of farmers [53]. Relatively stable market conditions are, therefore, characterized by narrower seasonal price variation and more consistent price transmission between farmers, collectors, and wholesalers.

In addition, wildlife damage is an important production challenge in forest‐based cultivation systems. Farmers commonly report losses caused by wild animals feeding on young shoots and developing capsules, particularly in areas adjacent to natural forests. To mitigate these losses, smallholder farmers use several traditional protection methods, including field guarding during fruiting stages, live fencing, buffer vegetation, and integration of korarima plots within managed agroforestry systems that reduce wildlife access [13, 51].

## 7. Conclusion

Korarima, an aromatic perennial endemic to Ethiopia, provides diverse benefits that extend from household kitchens to national and international markets. Its seeds and dried capsules enrich Ethiopian cuisine, adding distinctive flavor to sauces, breads, butter, and coffee, while various plant parts serve as traditional remedies for human and animal ailments. The spice also contributes to environmental protection by supporting forest conservation and preventing soil erosion in hilly landscapes. Despite these advantages, korarima production faces persistent obstacles, including limited agricultural inputs, weak market infrastructure, improper harvesting practices, pest pressure, and ongoing genetic erosion. Addressing these constraints requires a multifaceted approach. Priority actions include sustainable resource management, ex situ and in situ conservation of genetic resources, and the integration of indigenous knowledge into modern cultivation systems. Developing improved varieties, establishing clear quality standards, and promoting advanced technologies will be essential for enhancing productivity and ensuring that korarima remains a valuable economic and ecological resource for Ethiopia and beyond.

## Author Contributions

Netsanet Tena is the first author of the review article, whereas the coauthor has contributed equally to the literature collection, manuscript documentation, and its revision.

## Funding

This work was supported by Debre Berhan University, College of Agriculture and Natural Resource Sciences.

## Disclosure

The authors confirm that the content of the manuscript has not been published or submitted for publication elsewhere. All authors read and approved the final manuscript.

## Conflicts of Interest

The authors declare no conflicts of interest.

## Data Availability

No primary data were used to support this study.
